# Thermal variation associated stress response regulates the growth and reproductive potential of soybean looper

**DOI:** 10.1038/s41598-026-36978-1

**Published:** 2026-02-20

**Authors:** Rahul Debnath, Justin George, Rupesh Kariyat, Gadi V. P. Reddy

**Affiliations:** 1 USDA-ARS Southern Insect Management Research Unit, 141 Experiment Station Road, Stoneville, MS 38776 USA; 2 Department of Entomology and Plant Pathology, University of Arkansas, Fayetteville, AR 72701 USA

**Keywords:** Soybean looper, Antioxidant enzymes, Temperature stress, Life table, Pest management, Ecology, Ecology, Physiology, Plant sciences

## Abstract

The soybean looper (SBL) is one of the most damaging insect pests of soybean and other economically important crops worldwide. Although temperature has been reported to be a critical predictor of pest growth and development, very little is known about how temperature variations influence SBL population dynamics, which may aid in predicting SBL population outbreaks and dispersal. To examine this, we analysed the life table parameters of SBL by the age-stage, two-sex method under different temperature conditions. We also performed comparative analysis of antioxidant enzymes and total protein concentration from SBL larvae to unfold the enzymatic stress levels. Life table analysis revealed a significant extension in the developmental periods, and oviposition performance was adversely affected under extreme (extended) temperature conditions. Additionally, temperature stress elevated the antioxidant level, which negatively affected the growth and development of SBL, resulting in reduced plant damage. Population projections over an 80-day timescale simulation indicated that total SBL numbers would be significantly reduced during extreme temperature events compared to optimal temperatures. Overall, our findings suggest that extreme thermal stress had a negative effect on SBL growth and population progression, which could be used to meticulously predict SBL outbreaks and facilitate the development of a more efficient and sustainable management strategy.

**Supplementary Information:**

The online version contains supplementary material available at 10.1038/s41598-026-36978-1.

## Introduction

 Temperature is one of the most critical abiotic factors that can influence the development, reproduction, distribution, and migration of insect herbivores^1,2^. Insects, being ectothermic, are expected to be highly sensitive to changes in temperature. Through the course of evolution, insects have adapted to optimal temperature ranges that facilitate their growth and reproductive processes, enabling them to function efficiently^3,4^. However, the increasing frequency and duration of extreme climatic events are negatively affecting insect populations and their ability to disperse and thrive. Population dispersal serves as a crucial fitness trait in insects, which are likely subjected to significant selection pressure^5^. Simultaneously, the biodiversity of various globally important insect groups, such as pests, pollinators, and predators, has also been found to decline with prolonged heat exposure^6^. However, variation in temperature may impact the physiological traits and ecological interactions of most insect species^7^. On that account, to maintain ecological balance and resilience, it is essential to study the diversifying effects of varying temperature on insect species, including pests that cause severe damage to major crop species.

Soybean [*Glycine max* (L.) Merr.] is an economically important crop globally, and its production is heavily impacted by herbivory^8,9^. Insect herbivory has caused severe economic damage to the soybean crop in the United States, leading to yield losses exceeding 45%. Temperature fluctuations have been found to significantly influence seedling growth and development in soybean cultivars, with minimal interaction with photoperiod^10^. While infested by various insect orders, the soybean looper (SBL) (*Chrysodeixis includens* Walker) (Lepidoptera: Noctuidae) is widely considered as the primary pest of soybean^9,11,12^. Despite being a highly significant pest of soybean and other economically important crops, the influence of temperature regimes on SBL traits are unclear^13,14^. Although it is known that temperature fluctuations regulate the comparative performance and reproductive success of varying insect species^15–17^, whether temperature changes drive the stress enzyme activities, which in turn regulate the growth and reproductive outputs of SBL, is poorly understood.

Thermal stress is defined as the condition when temperature fluctuates outside the typical range, either low or high. Thermal stress in insects can lead to faster degeneration of reactive oxygen species (ROS), causing oxidative damage due to the disruption of their normal physiological processes^18,19^. Previous studies have shown that insect growth and development tend to be slower at lower^20^ and higher^21^ threshold temperatures. Since SBL exhibits migratory behavior under changing climatic conditions^22,23^, temperature fluctuations can assume a vital role in controlling their growth, physiology, and possible effects on soybean damage. To explore this hypothesis, we implemented a broad temperature gradient for assessing their detailed life cycle performance under laboratory circumstances. By contrast, there is very limited knowledge about how climatic thermal extremities regulate biological performance, demography, dispersion, as well as prey-predator interaction and status.

To address this knowledge gap, especially in the SBL-soybean system, we utilized an age-stage, two-sex life table to examine SBL life history traits, aiming to understand the survival and reproductive potential of SBL across five distinct temperature regimes (19, 23, 27, 31, and 35 °C). Additionally, this study presents the first report of how the stress enzyme levels influence biological traits and growth parameters of SBL across different temperatures under controlled conditions. Our specific objectives were to (i) employ an age-stage, two-sex life table approach to SBL at different temperature regimes, and to record the development time, longevity, and reproductive potential; (ii) explore antioxidant enzyme activities and protein content from the SBL culture maintained at different temperatures; (iii) determine the growth rate of SBL by feeding artificial diet at different temperature conditions; and (iv) evaluate projected population size/number over time. This comprehensive dataset will help to estimate temperature-driven phenology and population dynamics models based on specific natural atmospheric temperature conditions, enabling better planning for the effective implementation of an integrated management program for SBL.

## Materials and methods

### Insect culture

Soybean looper eggs were obtained from a commercial vendor (Benzon Research Inc., Pennsylvania, USA), and was reared on an artificial diet in the growth chamber (Percival Scientific Inc., Perry, Iowa, USA) under 27 °C, 60 ± 5% RH, and 12 L:12D conditions. Freshly laid eggs from the next generation SBL were used for further studies. Subsequent generations of SBL were reared on the artificial diet separately at each temperature regime (19 ± 1, 23 ± 1, 27 ± 1, 31 ± 1, and 35 ± 1 °C), 60 ± 5% RH, and 12 L:12D photoperiod were used to conduct an age-stage, two-sex life table study. In this study, we defined the lowest temperature condition as extreme low temperature (ELT, 19 °C) and the highest temperature condition as extreme high temperature (EHT, 35 °C) (hereafter, 19 °C: ELT, 35 °C: EHT). The feeding experiment and antioxidant enzyme assays were conducted by using separate cultures of soybean loopers maintained simultaneously at different experimental temperature settings. For this experiment, caterpillars were randomly selected individually from other colonies.

### Plant material

Soybean (variety 48XFO) seeds were planted and grown to the V3 stage in a greenhouse at the USDA-ARS facility located in Stoneville, MS, under 25 °C, 60% RH, and a 12 L:12D photoperiod. Plants were watered regularly, and fertilizers (Miracle-Gro^®^, The Scotts Company, US) were applied biweekly. Once soybean plants reached the third trifoliate leaf (vegetative, V3) stage, they were moved to the growth chambers (I36 VL) (Percival Scientific, Perry, IA) under specific experimental temperature conditions (60 ± 5% RH, and 12 L:12D). These plants were grown for two weeks in chambers to acclimatize them to different incubator conditions. After 2–3 weeks, soybean plants (with V7-V8 stage) were used for experiments (Fig. S1).

### Assessment of SBL survivability, developmental time, oviposition, and longevity at different temperature regimes to reveal the effect of extreme temperature conditions

**Fig. 1:**
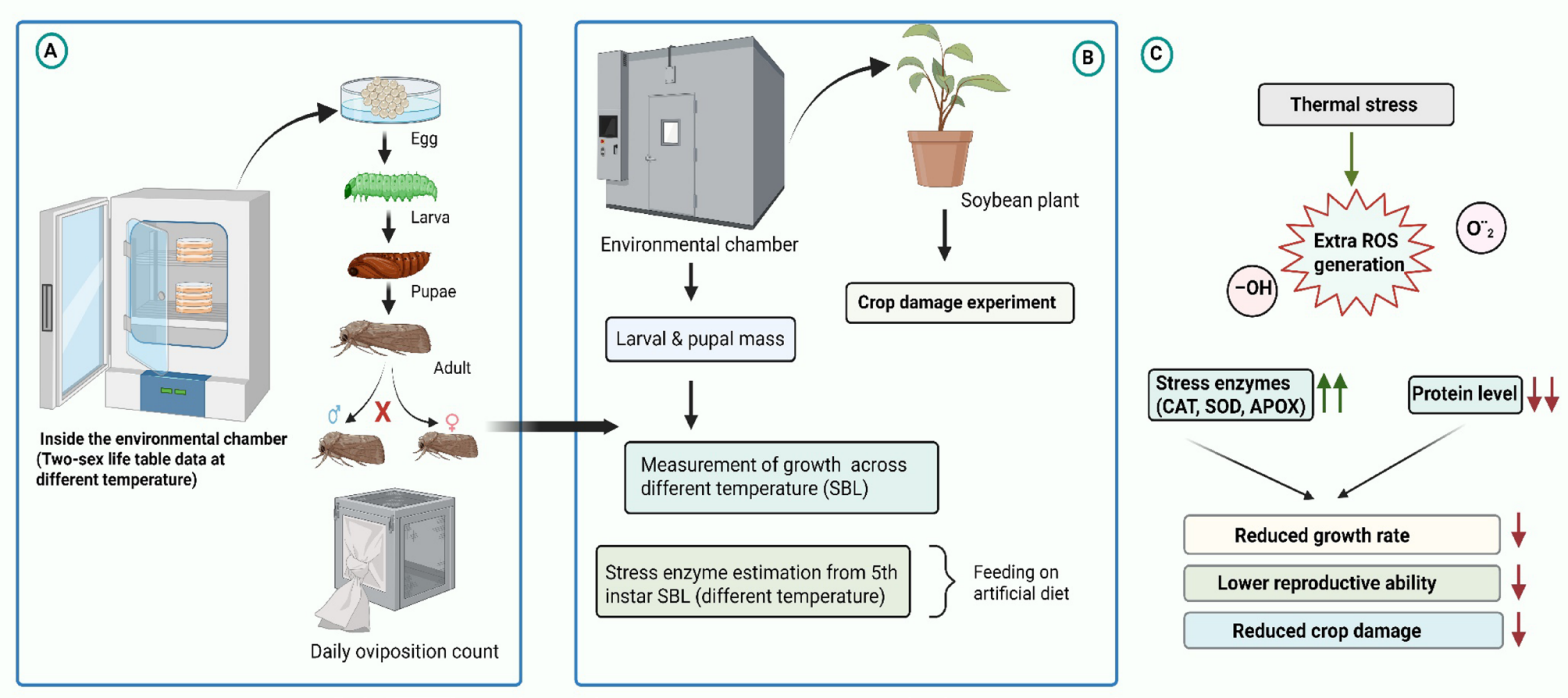
Schematic diagram illustrating the experimental setup and mechanism. (**A**) Two-sex life table analysis of SBL at different temperature regimes by feeding on an artificial diet. (**B**) Growth measurements and antioxidant enzymatic assays were performed on larvae of SBL, which were reared at specific experimental temperatures maintained inside an environmental chamber. (**C**) Thermal stress leads to the generation of excessive reactive oxygen species, resulting in the overexpression of antioxidant enzymes and a reduction in protein concentration. This suggests a potential biochemical process that impacts the growth and reproductive capabilities of SBL in thermal stress environments.

Pupae from the second-generation caterpillars were separated by sex based on morphological characteristics (the distal end of female pupae has a “V”-shaped separation, and males have a bump; both are microscopic) and transferred into individual mesh boxes (8.5 cm length × 12.5 cm width) for mating and oviposition. Then, freshly oviposited eggs (of the same age) were collected. Groups of one hundred eggs were collected randomly from different mating pairs and counted under a stereo zoom microscope (Leica S6E, Leica, United States). They were used to construct the age-stage, two-sex life table of SBL on an artificial diet at different temperature conditions. A total of 500 eggs were used in the life table study, with 100 eggs per temperature group (Fig. 1). Each egg served as a replicate for a specific temperature. Eggs were checked on a regular basis to record their hatching status.

Counted egg sheets were placed in petri dishes and kept in growth chambers at specific temperature settings. After hatching, the newly emerged first instar larvae were transferred individually into plastic cups with artificial diet using a soft-bristle brush. Each cup was equipped with a lid that had five small ventilation holes. Artificial diets were replaced every five days to ensure freshness. The presence of a head capsule and exuvium indicated a new developmental stage. Data on larval development stages, larval survival, pupal duration, and adult emergence were collected at every 24-hour intervals (Fig. 1). Following pupation, male and female pupae were separated and placed together in an oviposition box (8.5 cm length × 12.5 cm width) with fine mesh for oviposition. Eggs were counted daily, and the lifespan of adults was also recorded. Pupal and adult weights were taken from different SBL colonies reared at various temperatures.

### Measurement of SBL larval growth at different temperature regimes to evalute the developmental differences

Herbivore feeding and development on soybean plants under different temperature regimes were conducted using third-instar (3rd generation) larvae, which were reared on a standardized artificial diet in specific experimental temperature-controlled growth chambers. Larvae were chosen randomly from different batches. Four mature trifoliate leaves were selected on each plant, and an individual 3rd instar larva was allowed to feed on these leaves inside a meshed organza bag (Amazon, Seattle, WA, USA)^24^. Plants were watered every other day in the growth chambers to keep them healthy. Four larvae were allowed to feed on a single soybean plant; a total of five plants were used for individual temperature conditions (Fig. S1). The Mass gain percentage of SBL feeding on soybean leaves (V8 leaf stage) at specific temperature conditions was calculated by pre-weighing SBL larvae using an analytical balance (Sartorius, USA) before allowing them to feed. Weight gain of the individual larva was performed at 24-hour feeding intervals, and weight gain up to 120 h was recorded^25^. The formula used to calculate mass gain (%) is:

$${\rm \frac{Final\: mass\: - \: Initial \: mass}{Initial\: mass } \times 100}$$

### Determination of antioxidant enzymes in SBL to estimate oxidative stress level for thermal variation

Third-generation fifth instar SBL larvae fed on an artificial diet were used to determine enzymatic activity. Larvae were randomly collected in plastic cups from SBL colonies maintained in growth chambers under different temperatures and placed on ice for 5 min to slow them down, and larvae (*N* = 5) were dissected under a stereomicroscope (Leica S6E, Leica, United States). After dissection, the haemolymph was gently cleaned with distilled water. Midgut contents were homogenized in 1 mL of 10 mM NaCl. The solution was centrifuged at 12,000 rpm at 4 °C for 15 min. The supernatant was collected for enzymatic assays and stored at −20 °C (Thermo Scientific, TSX Series, United States) until used for analysis. Using this as an enzyme source, the activities of antioxidant enzymes, namely CAT (EC: 1.11.1.6), APOX (EC: 1.11.1.11), and SOD (EC: 1.15.1.1), were estimated using the procedure described^26–29^. Additionally, the total protein content was measured using the manufacturer’s protocol (Pierce Dilution-Free Rapid Gold BSA Protein Assay Kit, Thermo Scientific, Rockford, IL, USA) (supplementary materials).

### Population projection model to predict future estimated population size

Projected population growth of SBL under various temperature conditions was conducted using the methodology described^30,31,32^. The prediction of future population size and probable age-stage structure was performed using a specific computer program, TIMING-MS Chart^33^. The same data files generated by the age-stage, two-sex software were used for estimating population projections.

### Statistical analyses

The bootstrap technique, which is integrated into the TWOSEX-MS Chart program^32^, was employed to estimate mean and standard errors of the basic population parameters. To improve the reliability of the results, based on 100 original replications, 100,000 replications were applied in this study, and the data collected was checked for normal distribution. The paired bootstrap test^31^ was used to compare the differences at the 5% significance level for all the mentioned life table parameters. The differences in population parameters were also compared by using a paired bootstrap technique at the 5% level of significance. Damage analysis was conducted using the ordinal logistic regression model. The data’s normality was assessed using the Shapiro-Wilk test. Data on growth measurements of SBL on soybean leaves, and enzymatic activity of larvae across treatments were subjected to one-way analysis of variance (ANOVA) followed by Tukey’s test (HSD). All data were analysed using SPSS software (version 25.0) and GraphPad Prism (Version 8.0).

## Results

### Effect of temperature on survival, development, and longevity

 The mean developmental period and adult longevity are shown in Table S1. Incubation period of eggs was highest in extreme low temperature (ELT) of 19 °C (5.05 ± 0.08 days) and lowest in 27 °C (2.35 ± 0.05 days). The mean developmental duration of the larval stage increased significantly when reared at ‘ELT’ (17.60 ± 0.21 days) compared to 27 °C (12.37 ± 0.12 days) or 31 °C (12.68 ± 0.13 days) (Table S1). The pre-pupal duration was significantly lower in 27 °C (2.08 ± 0.08 days) than in other temperature conditions, and the time to pupation was also higher at ‘ELT’ (19 °C) and ‘EHT’ (35 °C) compared to 27 °C and 31 °C. The pre-adult durations were prolonged when the larvae were reared at ‘ELT’ (36.71 ± 0.35 days) than at comparatively higher temperatures of 27 °C (22.70 ± 0.16 days) and 31 °C (22.04 ± 0.23 days). Interestingly, the highest mean longevity of female insects was found in 27 °C (10.71 ± 0.18) and 31 °C (10.38 ± 0.22). Likewise, adult males also lived longer in 27 °C (8.33 ± 0.31 days) and 31 °C (8.04 ± 0.20 days) in comparison to ‘ELT’ (7.27 ± 0.26 days) (Table S1). Results showed that changes in temperature significantly impacted the developmental time, longevity, and survival rates. Both ‘ELT’ (19 °C) and ‘EHT’ (35 °C) resulted in a prolonged developmental period and decreased life span. Also, the effect of ‘ELT’ was more pronounced than ‘ETH’.

### Impact of temperature on demographic and reproductive potential

The impact of various temperatures on key demographic and reproductive potential is described (Table 1). The net reproductive rate (*R*_0_) was found to be highest at 27 °C and least in ‘ELT’ of 19 °C. Significantly lower net reproductive rate was observed at 23 °C and ‘EHT’ of 35 °C in comparison to 27 °C. Likewise, the finite rate of increase (λ) and the intrinsic rate of increase (*r*) were highest at 27 °C, while the lowest values were observed in ‘ELT’ or 19 °C (Table 1). On the contrary, mean generation time (*T*) showed an completely opposite trend; the longest time to complete a generation was observed at ‘ELT’ or 19 °C, and the shortest time was found in the case of 27 °C. The gross reproductive rate (*GRR*) was highest at 27 °C and lowest at ELT, with no significant differences observed between the other temperatures studied (Table 1).

**Table 1:** Impact of five different temperature regimes on reproductive and demographic parameters of SBL that fed on an artificial diet. Standard errors were estimated by using the bootstrap technique with 100,000 resamples. The difference was compared using the paired bootstrap test (*P* < 0.05). The means within a row followed by a different lowercase letter indicate significant differences between the treatments.

Parameters	19 °C	23 °C	27 °C	31 °C	35 °C
Intrinsic rate of increase, *r* (d^− 1^)	0.0960 ± 0.0068^a^	0.1372 ± 0.0060^b^	0.1965 ± 0.0048^c^	0.1754 ± 0.0059^d^	0.1205 ± 0.0063^b^
Finite rate of increase, *λ* (d^− 1^)	1.10 ± 0.01^a^	1.15 ± 0.01^b^	1.22 ± 0.01^c^	1.19 ± 0.01^d^	1.13 ± 0.01^b^
Net reproductive rate, *R*_0_ (offspring/individual)	54.79 ± 14.35^a^	111.05 ± 21.21^b^	240.27 ± 30.85^c^	160.93 ± 25.90^d^	75.81 ± 16.37^ab^
Mean generation time, *T* (d)	41.73 ± 0.74^a^	34.32 ± 0.46^b^	27.89 ± 0.17^c^	28.97 ± 0.25^d^	35.93 ± 0.42^e^
Gross reproductive rate, *GRR*	253.04 ± 69.28^a^	325.49 ± 58.89^ab^	419.77 ± 39.63^b^	312.36 ± 40.95^ab^	370.66 ± 92.71^ab^

**Fig. 2:**
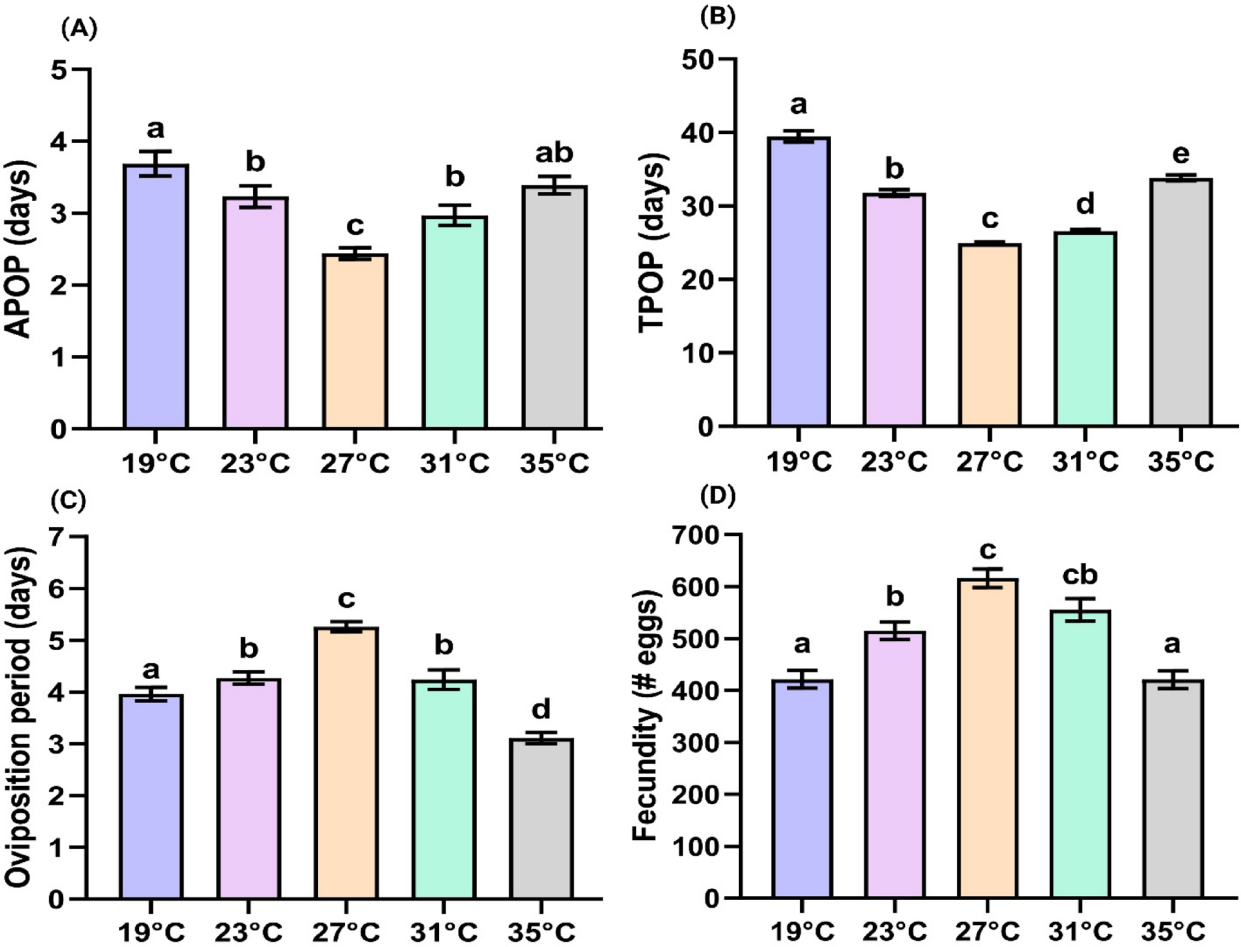
Ovipositional parameters (Mean ± SE) of SBL that fed on an artificial diet. (**A**) adult pre-oviposition period (APOP), (**B**) total pre-oviposition period (TPOP), (**C**) Oviposition period (days), (**D**) Fecundity (no. of eggs). Standard errors were estimated using 100,000 bootstrap resampling. Different letters indicate significant difference (paired bootstrap test at 5% significance level).

Changes in temperature also affected the oviposition parameters (Fig. 2). The female adult pre-oviposition period (APOP) was significantly (*P* < 0.05) decreased at 27 °C compared to ‘ELT’ or 19 °C and ‘EHT’ or 35 °C. Similarly, TPOP (total pre-oviposition period) also followed the same trend, and was significantly (*P* < 0.05) reduced at 27 °C in comparison to ‘ELT’ and ‘EHT’ (Fig. 2). Fecundity and the oviposition period were significantly (*P* < 0.05) increased when SBL was reared at 27 °C (~ 616 eggs/female) compared to ‘ELT’ or ‘EHT’ treatments (~ 421 eggs/female) (Fig. 2).

**Fig. 3:**
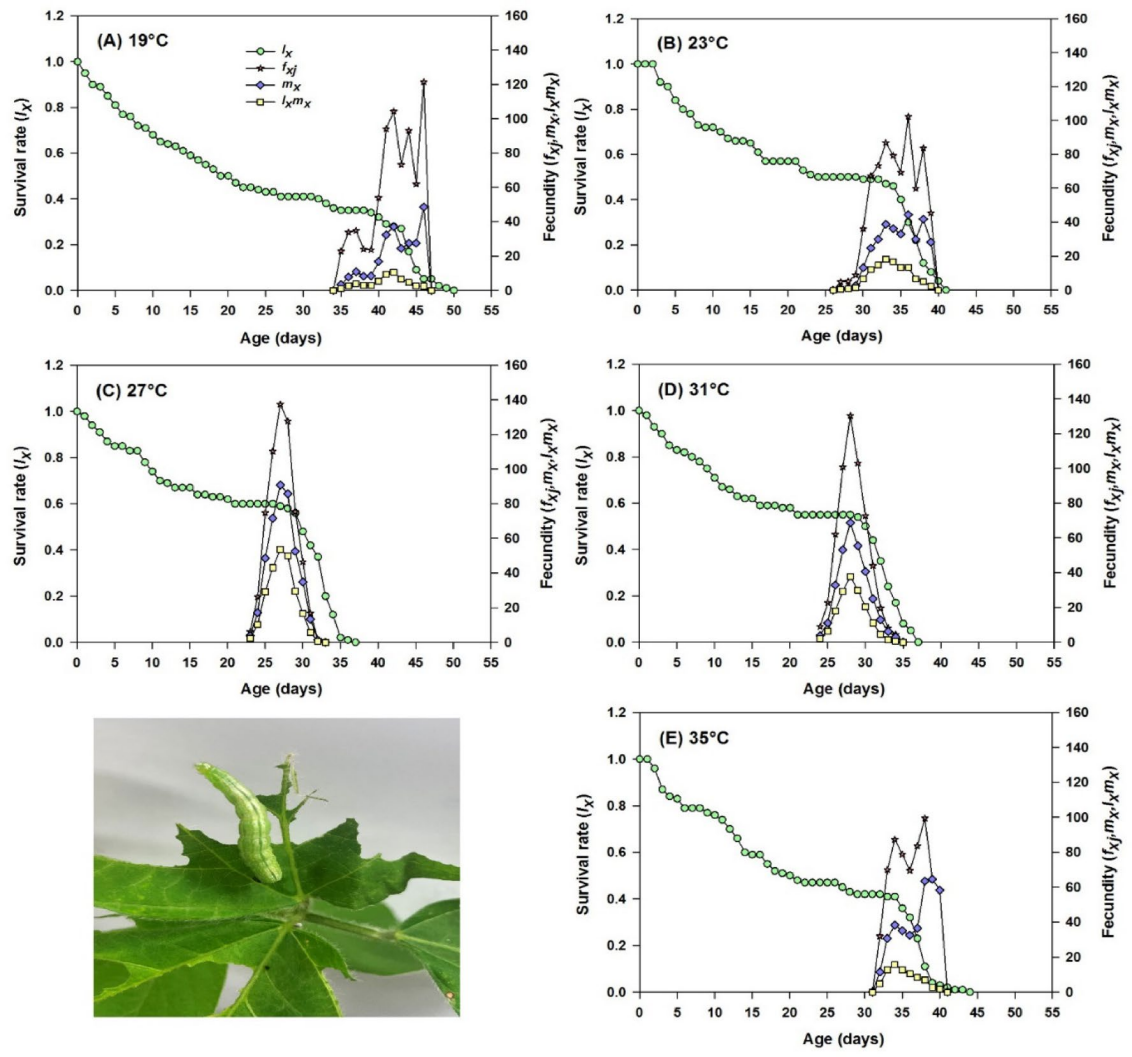
Age-specific survival rate (*l*_x_), age-stage specific fecundity (*f*_xj_), age-specific fecundity (*m*_x_), and age-specific maternity (*l*_x_*m*_x_) of *Chrysodeixis includens* (SBL) fed on artificial diet reared at five different temperature regimes (A-E).

The age-specific survival rate (*l*_x_), age-specific fecundity (*m*_x_), and age-specific maternity (*l*_x_*m*_x_) of SBL populations at different temperatures are shown in (Fig. 3). The *l*_x_ curves started to decline from 0.42 after 31 d in ‘EHT’, and 0.35 after 35 d in ‘ELT’, compared to 27 °C (0.6 after 23 d), where it was much delayed (Fig. 3A-E). The *m*_x_ and *l*_x_*m*_x_ curves showed that the reproduction started at 23 d and ended at 32 d for adults reared under 27 °C. In ‘ELT’, the reproduction began at the age of 35 d and ended at 46 d. However, for ‘EHT’, the reproduction started at 32 d and ended at 40 d. The daily oviposition rates were the highest at 27 °C compared to ‘ELT’ (Fig. 3A-E). These findings suggest that temperature stress significantly reduce both the fecundity and survival rates of SBL, indicating potential adverse effects on population dynamics.

**Fig. 4:**
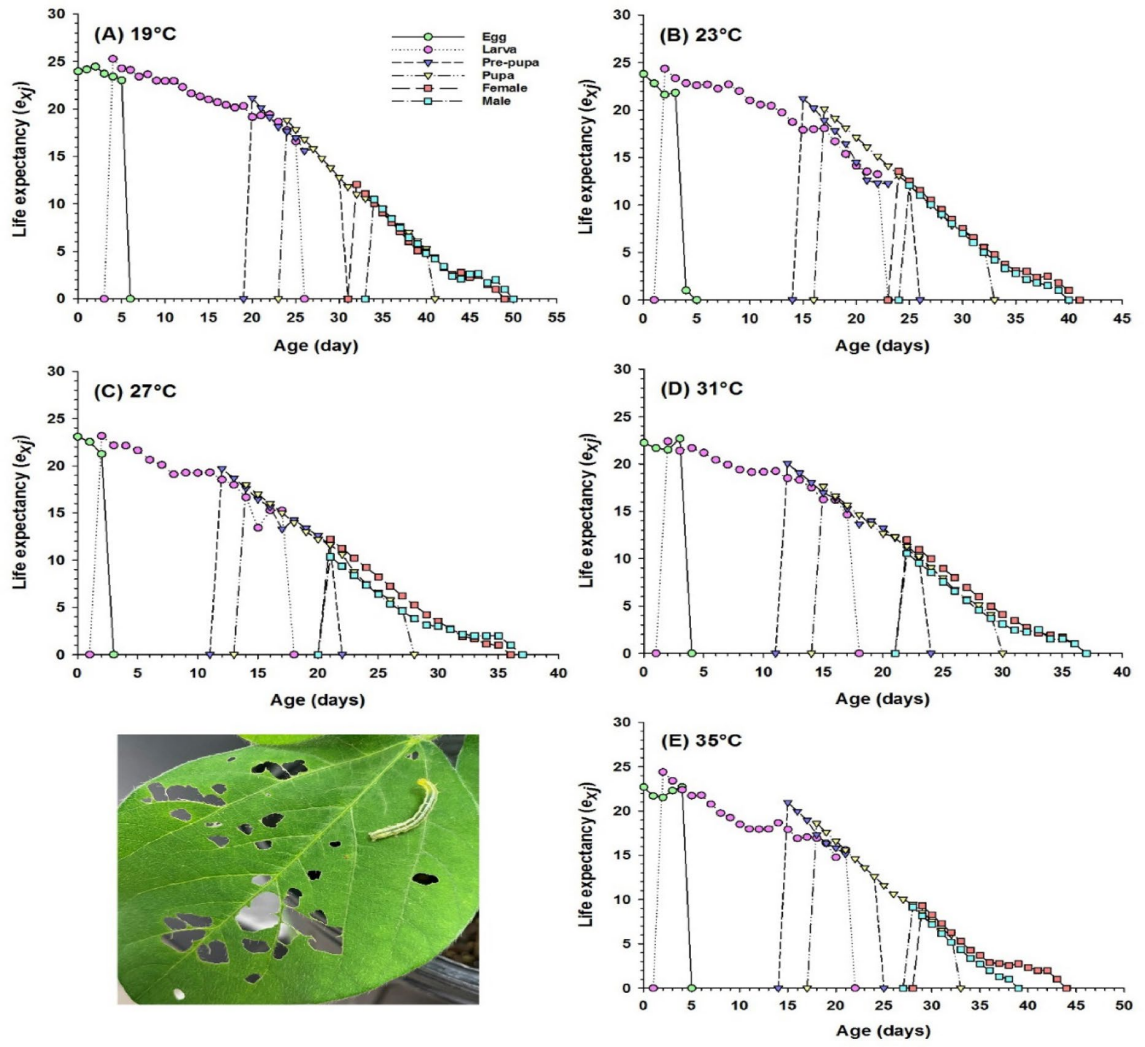
Age-stage-specific life expectancy (*e*_*xj*_) of *Chrysodeixis includens* (SBL) fed on an artificial diet reared at five different temperature regimes (A-E). General feeding damage caused by soybean looper on soybean leaves shown.

The age-stage survival rates (*S*_xj_) of SBL exhibited variations in developmental duration at different temperatures (Fig. S2). The survival rate was reduced, and the developmental period was delayed in the case of ‘ELT’ and ‘EHT’. On the other hand, longevity of adults was substantially higher at 27 °C and 31 °C (Fig. S2A-E). The age stage-specific life expectancy (*e*_xj_) depicts a longer life expectancy for adults at 27 °C and 31 °C compared to ‘ELT’ and ‘EHT’ (Fig. 4), and the age-stage reproductive value (*v*_xj_) represents the maximum reproductive potential (*V*_xj_ value) was achieved at 41 d (310 d^− 1^) and 33 d (304 d^− 1^) in ‘ELT’ and ‘EHT’ respectively, but it was 25 d (384 d^− 1^) at 27 °C (Fig. S3 A-E). This result demonstrates that exposure to extreme temperatures negatively affects survival rates, life expectancy, and reproductive potential.

### Growth regulation under various temperatures

**Fig. 5:**
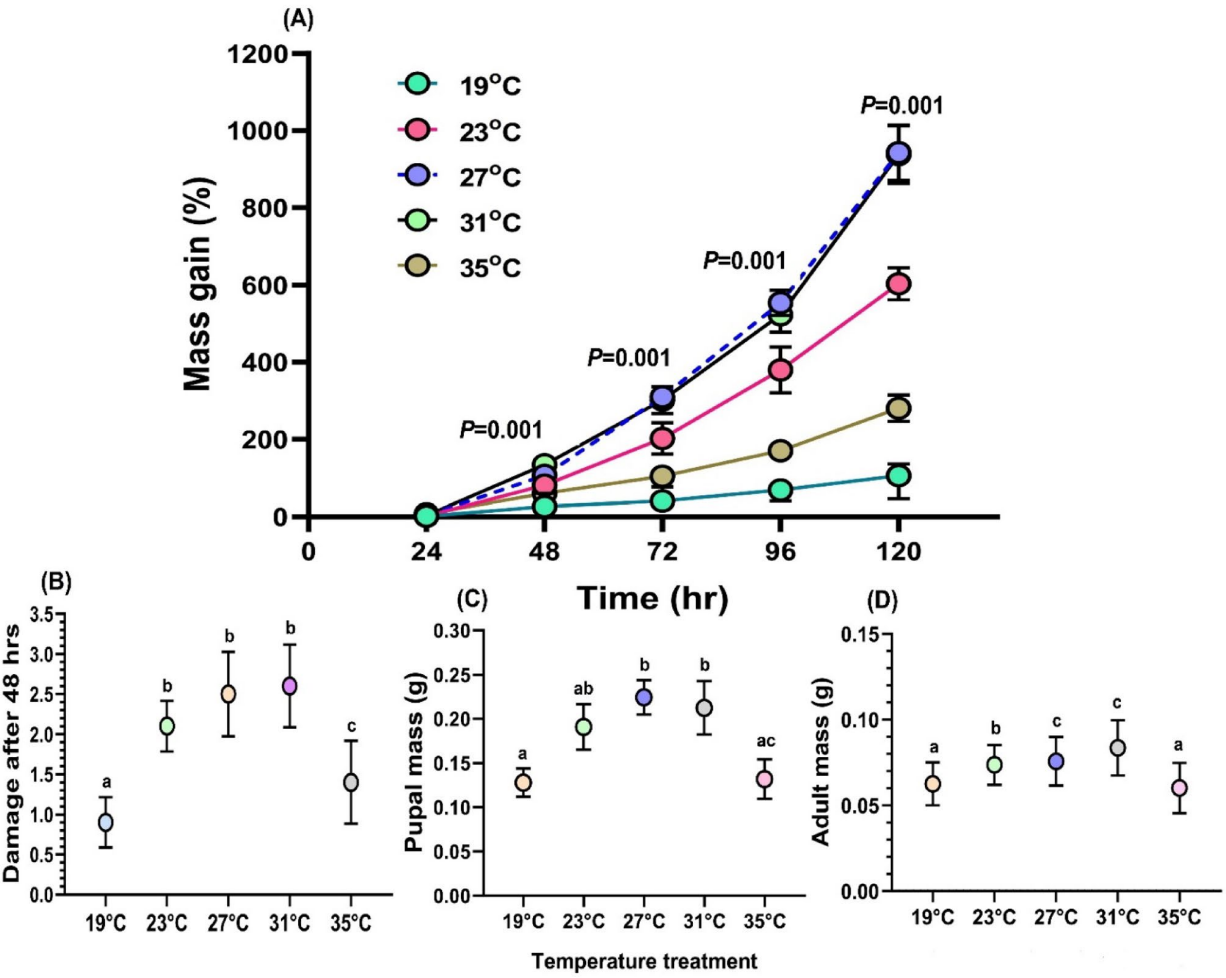
(**A**) Mass gain percentage of SBL larvae feeding on soybean plant at different temperature regimes. (**B**) Mean damage assessment of SBL larvae at various temperatures. (**C**) Mean mass (g) of SBL pupae when larvae were reared at different temperature regimes. (**D**) Mean mass (g) of SBL adults when larvae were reared at different temperature regimes.

No significant differences were observed in the larval mass gain following 24 h feeding on soybean plant leaves under different temperature regimes. After 48 h, a significant difference was noted in mass gain between 27 °C (106.99 ± 11.97%) and ‘ELT’ (26.87 ± 2.60%) (*F*_4,95_ = 27.27, *P* < 0.001). No significant differences were observed between ‘EHT’ (26.87 ± 2.60%), 23 °C (81.32 ± 4.74%) and 31 °C (134.53 ± 11.51%) (Fig. 5a). Statistical analysis for larval mass gain after 72 h showed significant differences between 27 °C (310.61 ± 18.40%) and ‘ELT’ (42.08 ± 2.88%) (*F*_4,95_ = 42.28, *P* < 0.001). However, no significant difference was found between ‘ELT’ and ‘EHT’ (104.71 ± 3.9%) (Fig. 5a). Similarly, after 96 h and 120 h, significantly higher mass gain was observed at 27 °C (554.10 ± 32.94% and 942.91 ± 72.04% respectively) in comparison to ‘ELT’ (73.56 ± 3.08% and 105.95 ± 4.11% respectively) and ‘EHT’ (170.51 ± 4.7% and 280.98 ± 7.71% respectively) (96 h: *F*_4,95_ = 68.94, *P* < 0.001, 120 h: *F*_4,95_ = 65.66, *P* < 0.001) (Fig. 5a). No significant difference found between 27 °C and 31 °C.

Pupal and adult mass at different temperature conditions following pupation was also calculated. Significant differences were observed in the pupal mass between different temperature treatments (*F*_4,95_ = 76.85, *P* < 0.001) (Fig. 5c). Increased pupal mass was observed at 27 °C (0.227 ± 0.007 gm) and 31 °C (0.212 ± 0.011 gm) compared to ‘ELT’ (0.129 ± 0.004 gm) and ‘EHT’ (0.127 ± 0.008 gm) (Fig. 5c). Mass analysis of the adult SBL (1:1 male-female) reared under different temperature conditions showed significant differences (*F*_4,95_ = 9.751, *P* < 0.0001) between temperature treatments. Adult mass was highest in 27 °C (0.076 ± 0.003 gm) and lowest in ‘EHT’ (0.060 ± 0.03 gm) (Fig. 5b). Damage scale analysis reported that SBL larvae are capable of the least damage in ‘ELT’ (X = 0.90 ± 0.10) compared to other temperatures. Also, no significant differences were observed between 23 °C, 27 °C, and 31 °C (Fig. 5 d).

### Effect of temperature on the enzymatic activity of SBL larvae

**Fig. 6:**
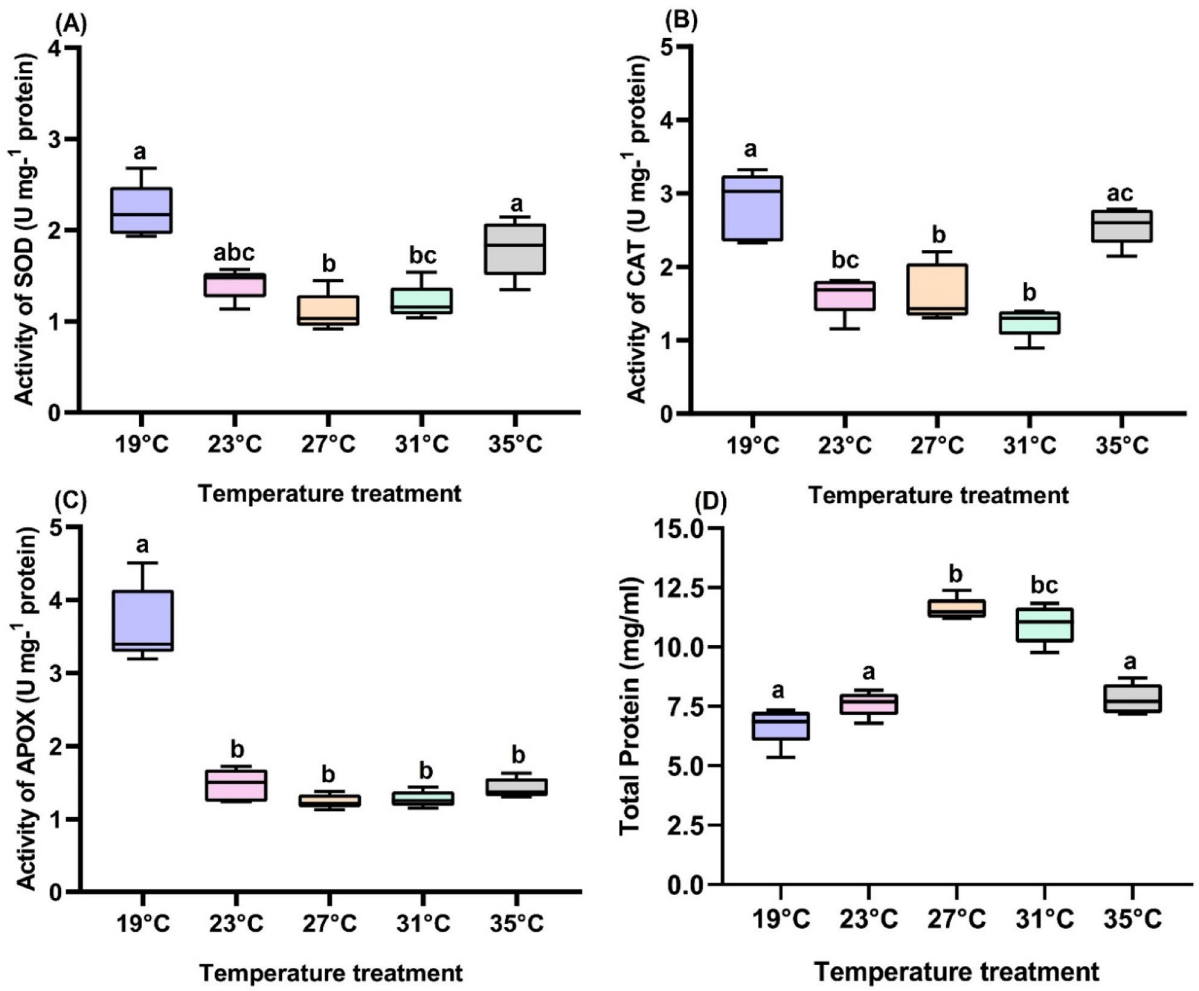
Effects of temperature regimes on antioxidative enzymes and total protein content in 5th instar SBL larvae. (**A**) Superoxide dismutase (SOD), (**B**) catalase (CAT), (**C**) ascorbate peroxidase (APOX), (**D**) total protein content. Different letters in treatments indicate significant differences at the 5% level of significance.

**Fig. 7:**
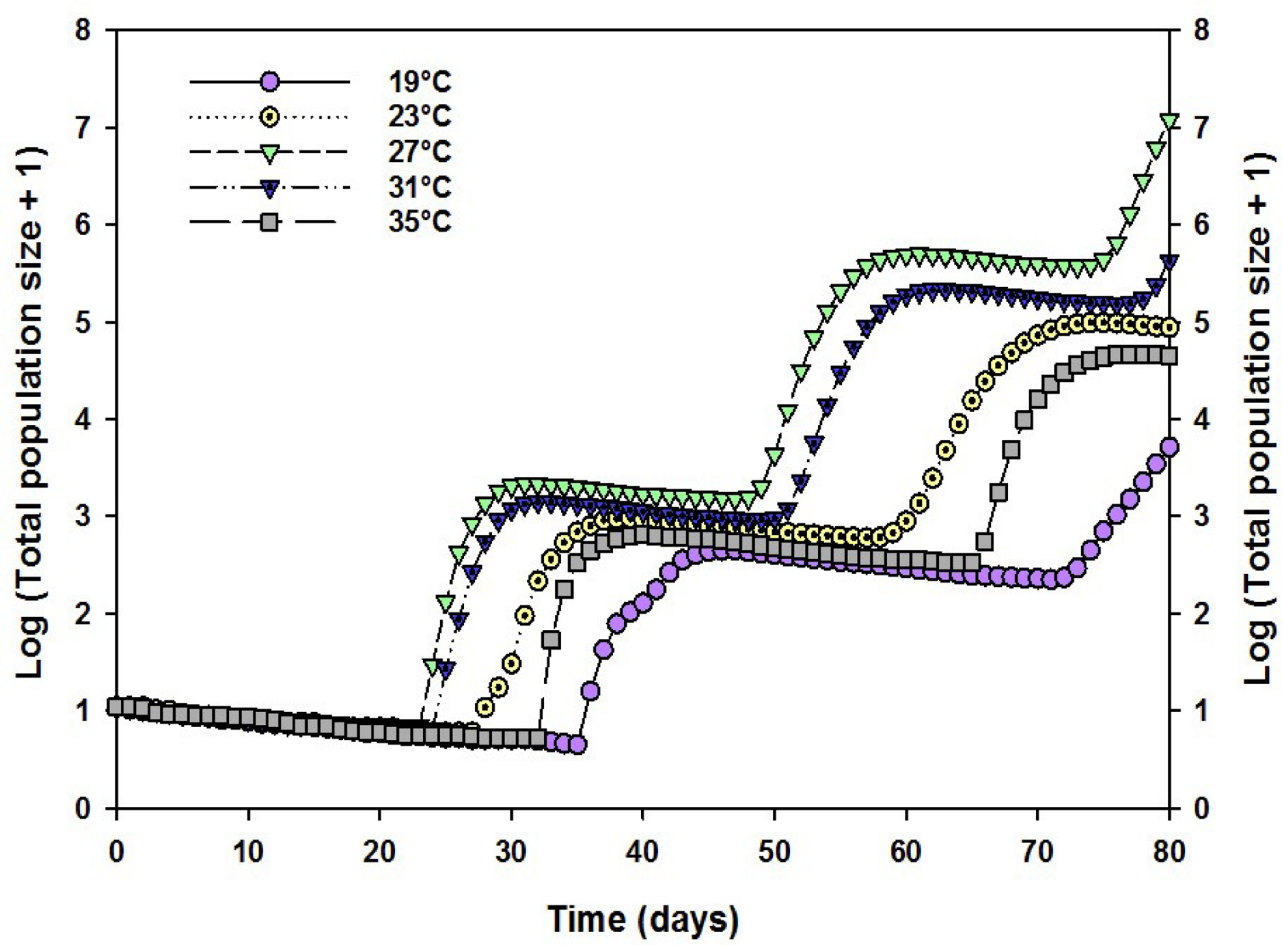
Population projections of *Chrysodeixis includens* (SBL) fed on an artificial diet reared at five different temperature regimes from an initial cohort of 10 eggs at 80 days.

The effect of temperature regimes on antioxidant enzymes (SOD, CAT and APOX) and total protein concentration of 5th instar SBL larvae was also studied (Fig. 6). SOD enzyme activity at different temperatures were regulated significantly (*F*_4,20_ = 231.3, *P* < 0.001), and a 2-fold increase in enzyme activity was observed at ‘ELT’, and a 1.63-fold in ‘EHT’ than at 27 °C (Fig. 6a). CAT and APOX enzyme activity was enhanced 1.73 and 2.94- fold respectively in ‘ELT’ in comparison to 27 °C, and 2.27-fold and 2.87-fold compared to 31 °C (Fig. 6b, c). Total protein concentration under stressed conditions like ‘ELT’ and ‘EHT’ had decreased 1.72 and 1.49-fold compared to 27 °C (Fig. 7d).

### Impact of temperature on population projection

The projected growth of the SBL population that was maintained at various temperatures, simulated from a starting population of 10 larvae using the TIMING-MS Chart program, is reported. The SBL population, maintained at various temperatures, was simulated from a starting population of 10 larvae using the TIMING-MS Chart program (Fig. 7, Fig. S4). The total population size of SBL was projected to be approximately 245,051 individuals (166,728 female, 78,323 male) at 27 °C after 80 days. The population size of ‘ELT’ was projected to be the smallest (~ 5151 individuals) among all the other temperature treatments. ‘EHT’ also exhibited a decreased trend in population size that was estimated to be nearly ~ 45,177 individuals (Fig. 7).

### Relationships between life table parameters and antioxidant enzymes of SBL

**Fig. 8:**
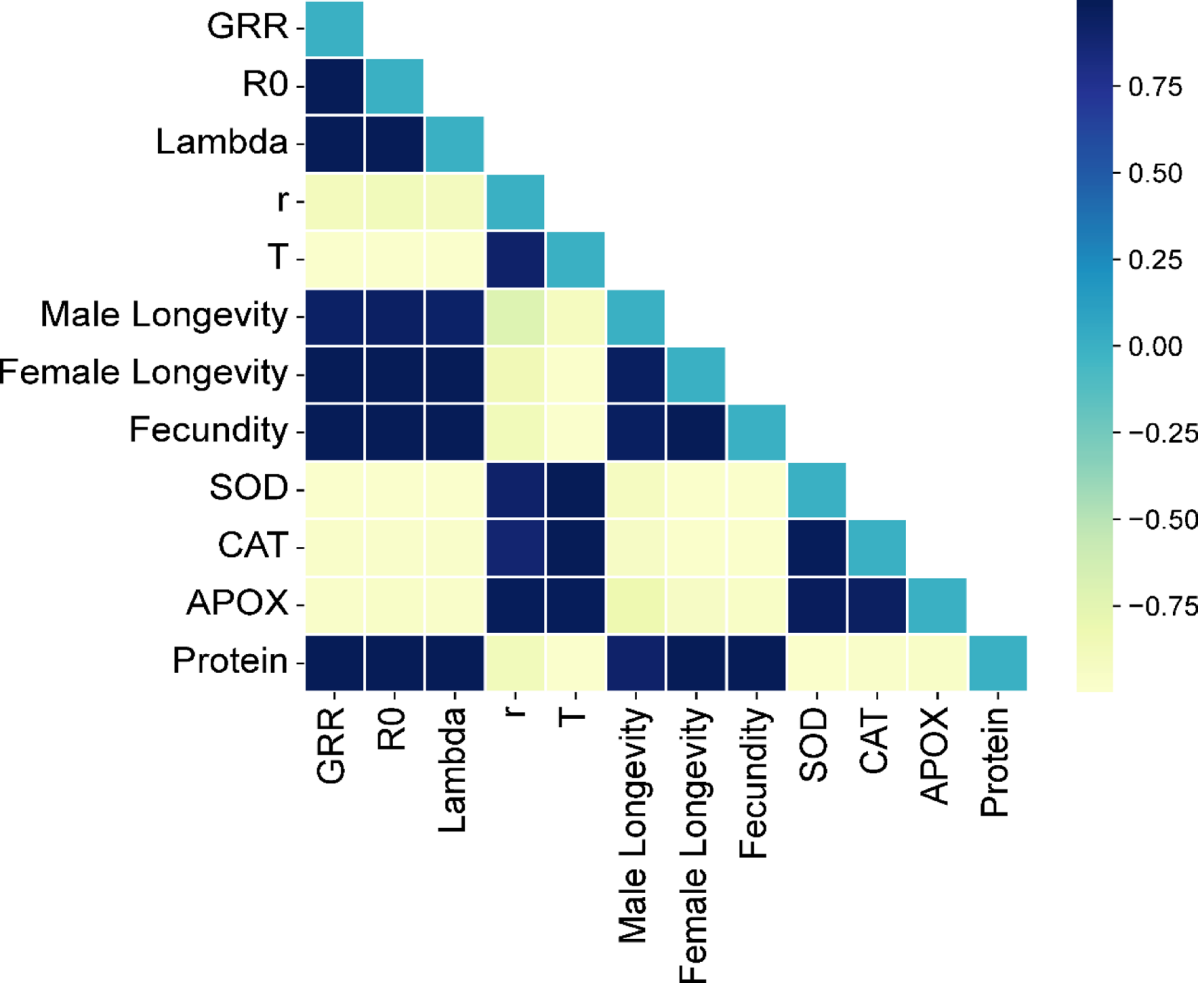
Correlation coefficient plot showing the relationship between SBL reproductive and demographic parameters and antioxidant enzyme activities of SBL larvae at different temperature regimes.

 The Pearson correlation of coefficient analysis revealed that the life table and reproductive parameters of SBL were significantly (*P* < 0.001) correlated with plant and larval enzyme properties (Fig. 8). The main reproductive parameters *GRR*, *R*_o_ and *r* of SBL at different temperatures was negatively correlated with larval stress enzyme level (Table S2). Contrarily, mean generation time (*T*) had negatively correlated with larval protein level (*r* = 0.99, *P* < 0.001) (Fig. 8). Nevertheless, male and female longevity were also found to be negatively correlated with stress enzyme levels and positively correlated with total larval protein levels. Similarly, fecundity was found positively correlated with total larval protein (*r* = 0.98, *P* < 0.001) and negatively correlated with stress enzyme level (Fig. 8, Table S2).

## Discussion

Temperature variation throughout the year influences crop health and, consequently, the performance of insect herbivores. In this study, we investigated how temperature regimes regulate the stress physiology of SBL, influencing their life history traits, reproductive potential, and their ability to damage soybeans under varying temperature conditions. We observed that SBL performance was significantly inferior at both extreme temperatures (cold and warm), causing reduced oviposition, delayed growth, and lower damage compared to optimal temperature conditions. Intriguingly, temperature stress during the larval stage was characterized by low protein levels and elevated stress enzyme accumulation, which negatively impacted the performance of SBL. Our research reveals that for SBL, adapting to environmental stressors demands more energy, ultimately affecting their overall performance.

Implementation of effective pest management strategies requires accurate knowledge of pest phenology, development, and the intensity of crop damage they cause under varying temperatures. To this end, we performed an age-stage two-sex life table experiment using SBL at different temperatures. We observed that egg hatching time and the duration of immature larval stages were significantly longer at extreme low temperatures (ELT: 19 °C), slightly reduced at 23 °C, and most efficient at 27 °C. The duration of pupal and pre-adult stages was significantly longer at extreme high temperature (EHT: 35 °C) compared to 27 °C and 31 °C. This observation aligns with previous studies, which reported that SBL requires an extended period for the development of its immature stages, and showed low population densities below 19.7 °C, and the optimal development was observed at 25.4°C^34^. In our preliminary studies, we had tested the SBL larval development at temperatures ranging from 10 °C to 40 °C. However, eggs below 19 °C and above 35 °C either failed to hatch or couldn’t complete the larval stages. This observation strongly correlates with the study of Tingle & Mitchell (1977)^35^, who reported that larval development and reproduction of SBL could only be achieved at 16 °C or above. Moreover, the growth and developmental trends of SBL have changed due to temperature variations, clearly demonstrating why understanding the optimal temperature is crucial for effective SBL management.

In addition, adult lifespan was found to be longer at 27 °C compared to the extreme temperatures we investigated in this study. A similar effect of temperature on the adult lifespan of lepidopteran species has been previously reported^36–38^. Additionally, studies have also reported that temperature fluctuations can regulate the duration of instars, longevity, and oviposition capability of adult insects^16,17^. Similar results from our research also indicate that temperature variations can significantly impact the development, survival, and adult longevity of SBL. The extreme low temperature (ELT: 19 °C) treatment caused the most significant harm to the larval stages, leading to substantial growth delays and inferior reproductive ability. However, studies have reported that under various stress conditions (biotic or abiotic), developmental delay leading to lifespan extension is a common occurrence^39–41^. This could be due to the fact that temperature changes lead to changes in metabolism, food intake capacity, and energy allocation^17^. Thus, it is crucial to understand that the energy produced by anabolic and catabolic metabolic processes, which are vital for insect growth and reproduction, is influenced by variations in environmental temperatures.

Prolonged exposure to thermal stress (both cold and heat) leads to oxidative stress resulting from the excessive production of reactive oxygen species (ROS)^42^ (Fig. 1). These elevated levels of ROS are mitigated by vital antioxidant enzymes, including SOD, CAT and APOX. Antioxidant enzymes are crucial for cellular adaptation, with SOD converting superoxide radicals into hydrogen peroxide (H_2_O_2_), which is further metabolized by CAT within the cell^29,43,44^. In this study, significantly elevated levels of SOD, CAT, and APOX enzyme activity in larval haemocytes were observed under extreme conditions, indicating their role in defending against thermal stress. Similar results were observed in the increased levels of SOD and CAT enzyme activities under thermal stress conditions at both low and high temperatures in *Mythimna separata* (Lepidoptera: Noctuidae)^45^. Another study by Nabizadeh & Kumar (2011)^46^ followed the same phenomenon: under thermal stress conditions, antioxidant enzyme activity increased in *Bombyx mori* (Lepidoptera: Bombycidae). Additionally, excessive levels of ROS accumulation can cause cellular damage to proteins, lipids, and nucleic acids, ultimately leading to cell death and impacting insect performance and survival ability^47,48^. We also found a decrease in protein levels in larvae exposed to extreme temperature conditions. Singh et al. (2013)^49^ reported that exposure to low temperatures in silkworm larvae resulted in a decrease in total protein levels. These research findings clearly indicate that thermal stress negatively impacts the physiological and biochemical adaptations of SBL, leading to a reduction in their fitness. Further research is needed to quantify ROS levels and analyse the impact of these changes on specific heat shock protein (*hsp*) expression in SBL, examining both mRNA and protein levels. Nevertheless, our study did not account for how stress enzyme properties of soybean plants affect pest performance, which presents a compelling direction for future investigation of this current research.

The age-stage, two-sex life table is different from traditional life tables and can eliminate many of the inherent error characteristics of female-based life tables^50–52^. Findings from this study indicated that optimum temperature conditions significantly increased the key reproductive parameters, including the intrinsic rate of increase (*r*_*m*_), finite rate of increase (*λ*), net reproductive rate (*R*_*0*_), and fecundity, compared to thermal stress conditions (ELT and EHT). Thermal extremes, both cold and heat, had negative impacts on the overall development and survivability of SBL. Our results indicate that the oviposition potential of SBL improves when the life cycle is performed at the optimum temperature. On the other hand, female moths oviposit comparatively fewer eggs in cold and hot temperature conditions. This could be attributed to the slowdown of the metabolism rate of insects in abiotic (temperature) stress conditions. Another possible explanation could be that the long term exposure to environmental stress in insects can trigger the production of defensive chemicals, but it can also weaken their immune system by diverting energy away from the heat shock response^53^. However, population projection also predicted that under 27 °C temperature conditions, SBL population size will be maximized among all the experimental temperature regimes studied. In contrast, the expected population number decreased significantly in extreme temperature conditions compared to optimum temperature. These findings align with other research studies indicating that thermal stress contributes to insect population decline. Therefore, it is plausible that higher mortality and growth retardation in extreme environmental conditions can lead to a decline in population.

Results from the growth experiment revealed significant differences in larval growth between different temperature regimes from the beginning, starting at 24- to 120-hour time periods. Larvae gained considerably less mass when reared at extreme temperatures compared to optimum temperatures. Leaf damage by SBL larvae at 27 and 31 °C was found to be comparatively higher than the temperature stress conditions. Likewise, pupae and adults that emerged from extreme temperatures had lower body mass than those under optimal conditions. Most importantly, the results of our study showed that extreme warm and cool temperatures both have negative impacts on insect growth and reproduction. Based on our results, it’s clear that whether it is cold or hot, extreme thermal conditions have an adverse impact on SBL. These observations complement previous findings that extreme temperatures are responsible for slowing down the development process in various insect species^16,17^. The distribution of thermal responses is uneven, leading to variations in life-stage timing and population dynamics of different insect species^54,55,56^. Since climate change is causing a rise in atmospheric temperatures, it may lead to population unpredictability in insects and potentially modify their adaptation to the environment. The findings of this study will enable predictions about the future expansion, outbreaks, and associated demographic changes in SBL, especially in light of climate change and the rise in temperature.

## Conclusion

In conclusion, this present study has demonstrated that SBL can develop and reproduce across a wide temperature range from 19 °C to 35 °C. Thermal stress during the larval development contributes to oxidative stress, and protein breakdown may occur, leading to lower protein levels at extreme temperatures compared to the optimum temperature. Our findings that thermal stress conditions, both cold and heat, negatively affect the larval development and adult oviposition capacity as well as population projection show that more severe thermal events predicted for the future by climate change scenarios will have adverse effects on the population size of SBL. This information will help simulate population dynamics, determine population occurrences and peaks, and ultimately develop efficient management strategies to control SBL. Further research needs to prioritize recognizing the specific heat shock proteins and understanding the functional genetic changes involved in regulating key biological parameters during thermal stress.

## Supplementary Information

Below is the link to the electronic supplementary material.

Supplementary Material 1

## Data Availability

All data is provided within the article, and any additional information is available upon request.
