# Supplementary material for: Thermal variation associated stress response regulates the growth and reproductive potential of soybean looper
Source: Sci Rep. 2026 Feb 20;16:9976. doi: 10.1038/s41598-026-36978-1 (PMC13021961; doi:10.1038/s41598-026-36978-1)
Supplement: Supplementary file 1 — Supplementary Material 1 [file 41598_2026_36978_MOESM1_ESM.docx]

# Supplementary Materials

**Details protocol of enzymatic antioxidants estimation from the leaf :**

Superoxide dismutase (SOD) activity was estimated according to the method of Giannopolitis and Ries (1977). The enzyme was extracted by homogenizing plant tissue (1 g) in prechilled 10 mL of 100 mM Na-phosphate buffer (pH 6.8) containing 1% w/v PVPP and centrifuged at 6000 rpm at 4 °C for 10 min. The supernatant was used as an enzyme source, and the enzyme activity was determined by measuring the photochemical reduction ability of nitroblue tetrazolium chloride (NBT). The reaction mixture contained 0.05 M Na2CO3, 0.1 mM EDTA, 63 mM NBT, and 13 mM riboflavin. Riboflavin was added last. The assay mixture was placed under a 40 W fluorescent lamp at a distance of 30 cm ,and maintained at 25 °C for 30 min. Absorbance was then measured at 560 nm against the non-irradiated sample using a UV–visible spectrophotometer (Cary 60 UV-VIS, Agilent). The enzyme activity was expressed as units g-1 dry weight leaf tissue/min.

Ascorbate peroxidase (APOX) activity was determined according to Nakano and Asada (1981). The assay mixture consisted of 1 mL of enzyme extract (prepared from 1 g of treated leaf tissue), 0.5 mM ascorbic acid, and 0.1 mM EDTA. Parallel experiments in the presence of p-chloromercuribenzoate (50 M) were performed to rule out any interference from guaiacol peroxidases. Absorbance was measured at a wavelength of 265 nm in the UV–visible spectrophotometer (Cary 60 UV-VIS, Agilent), and the activity of APOX was expressed as Unit g−1 dry weight leaf tissue/min.

Catalase (CAT) was estimated using the procedure described by Snell and Snell (1971). Treated plant tissues (500 mg) were homogenized in a cold mortar with a pestle in 0.1 M sodium phosphate buffer (pH 7.0) containing 1% polyvinylpolypyrrolidone (PVPP), and the homogenate was centrifuged at 5000 rpm for 10 min at 4 °C. The supernatant was selected for enzyme assays. One mL of enzyme extract was added to 1 mL of 0.5 mM H2O2 followed by incubation for 15 min at 37 °C. The reaction was stopped by adding 2 mL of 1% TiSO4 in 25% H2SO4. The assay mixture was centrifuged again at 5000 rpm, and the absorbance of the supernatant was measured at 420 nm using the UV–visible spectrophotometer (Cary 60 UV-VIS, Agilent). The enzyme activity was expressed as Unit g−1 dry weight leaf tissue min−1 according to Fick and Qualset (1975).


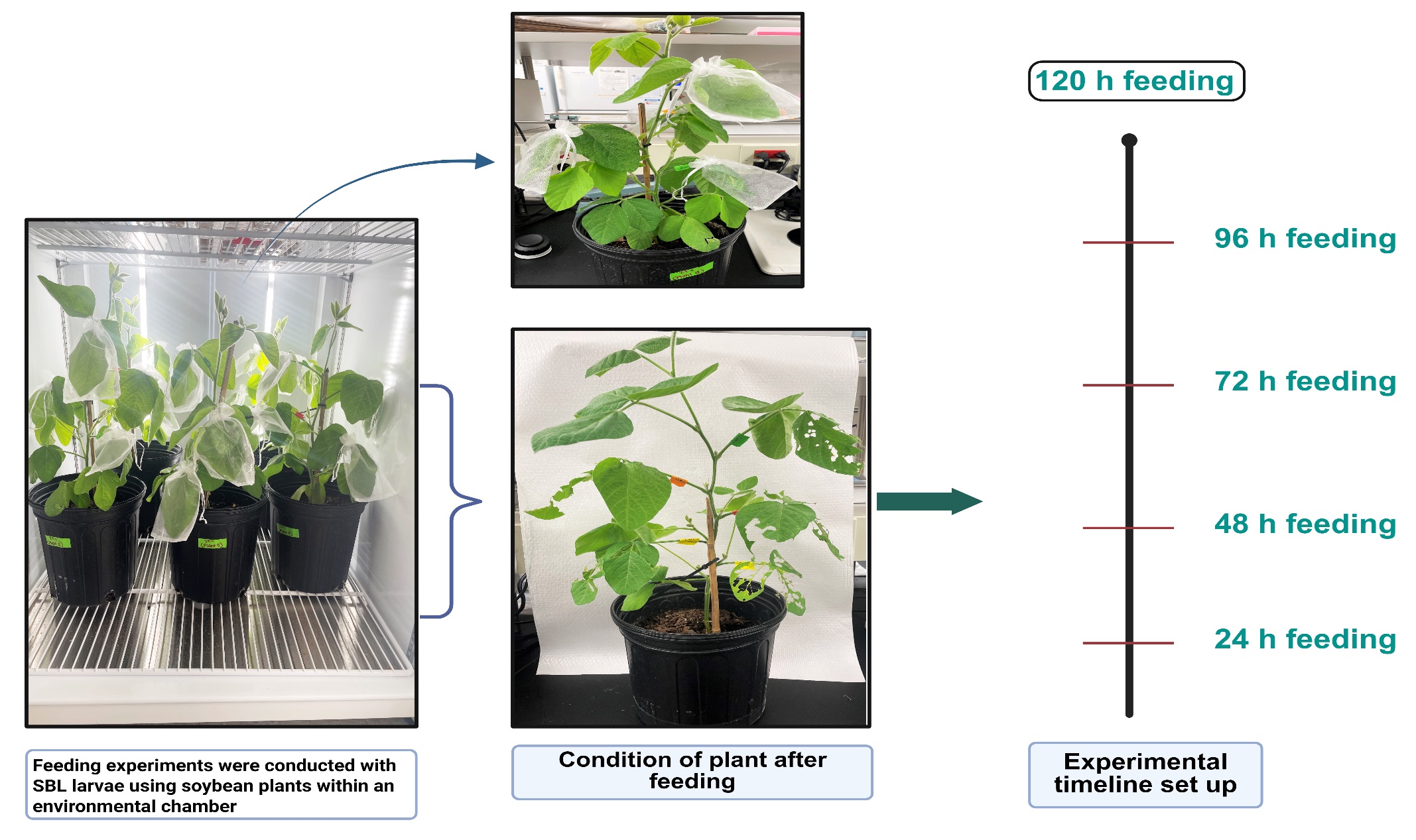


**Fig 1.** Schematic diagram illustrating the experimental setup for feeding SBL larvae and the resulting consequences.

**Table 1.** Development time and longevity of adults (Mean ± SE) of *Chrysodeixis includens* fed on an artificial diet at five different temperature regimes. Standard errors were estimated using 100,000 bootstrap resampling. Data followed by the different lower-case letters within the row were significantly different based on a paired bootstrap test.

|  | 19 °C | | 23 °C | | 27 °C | | 31 °C | | 35 °C | |
| --- | --- | --- | --- | --- | --- | --- | --- | --- | --- | --- |
| Parameters (days) | n | Duration (days) | n | Duration (days) | n | Duration (days) | n | Duration (days) | n | Duration (days) |
| Egg | 79 | 5.05 ± 0.08a | 88 | 3.38 ± 0.07b | 91 | 2.35 ± 0.05c | 90 | 2.73 ± 0.06d | 84 | 3.81 ± 0.08e |
| Total larval duration | 45 | 17.60 ± 0.21a | 57 | 15.19 ± 0.21b | 65 | 12.37 ± 0.12c | 60 | 12.68 ± 0.13c | 50 | 15.56 ± 0.17b |
| Prepupal duration | 41 | 4.32 ± 0.07a | 50 | 2.68 ± 0.09b | 63 | 2.08 ± 0.08b | 58 | 2.36 ± 0.15cb | 47 | 2.96 ± 0.07b |
| Pupal duration | 35 | 9.51 ± 0.19a | 49 | 7.67 ± 0.83b | 60 | 6.15 ± 0.05c | 55 | 6.40 ± 0.08d | 42 | 8.36 ± 0.15e |
| Preadult duration | 35 | 36.71 ± 0.35a | 49 | 28.94 ± 0.30b | 60 | 22.70 ± 0.16c | 55 | 22.04 ± 0.23d | 42 | 30.64 ± 0.23e |
| Adult longevity (days) | | | | | | | | | | |
| Female | 13 | 8.38 ± 0.18a | 22 | 9.00 ± 0.24b | 39 | 10.71 ± 0.18c | 29 | 10.38 ± 0.22c | 18 | 7.83 ± 0.26d |
| Male | 22 | 7.27 ± 0.26a | 27 | 7.77 ± 0.17ab | 21 | 8.33 ± 0.31b | 26 | 8.04 ± 0.20b | 24 | 6.38 ± 0.18c |

**Table 2.** Pearson correlation coefficients (r) of reproductive parameters of *Chrysodeixis includens* and antioxidant enzyme properties.

| Parameters | SOD (Leaf) | | CAT (Leaf) | | APOX (Leaf) | | Protein (Leaf) | | SOD (Larvae) | | CAT (Larvae) | | APOX (Larvae) | | Protein (Larvae) | |
| --- | --- | --- | --- | --- | --- | --- | --- | --- | --- | --- | --- | --- | --- | --- | --- | --- |
|  | *r* | *P*_value_ | *r* | *P*_value_ | *r* | *P*_value_ | *r* | *P*_value_ | *r* | *P*_value_ | *r* | *P*_value_ | *r* | *P*_value_ | *r* | *P*_value_ |
| *GRR* | -0.803 | 0.0001 | -0.570 | 0.003 | -0.625 | 0.001 | 0.423 | 0.035 | -0.743 | 0.0001 | -0.590 | 0.002 | -0.578 | 0.002 | 0.756 | 0.0001 |
| *r_m_* | 0.280 | 0.175 | 0.257 | 0.214 | 0.514 | 0.005 | -0.254 | 0.221 | 0.609 | 0.001 | 0.507 | 0.010 | 0.863 | 0.0001 | -0.360 | 0.077 |
| *λ* | -0.857 | 0.0001 | -0.642 | 0.001 | -0.790 | 0.0001 | 0.544 | 0.005 | -0.809 | 0.0001 | -0.709 | 0.0001 | -0.679 | 0.0001 | 0.934 | 0.0001 |
| *R*_0_ | -0.882 | 0.0001 | -0.672 | 0.0001 | -0.722 | 0.0001 | 0.557 | 0.004 | -0.761 | 0.0001 | -0.661 | 0.0001 | -0.585 | 0.002 | 0.904 | 0.0001 |
| *T* | 0.844 | 0.0001 | 0.691 | 0.0001 | 0.832 | 0.0001 | -0.547 | 0.005 | 0.865 | 0.0001 | 0.791 | 0.0001 | 0.816 | 0.0001 | -0.360 | 0.077 |
| Fecundity | -0.951 | 0.0001 | -0.575 | 0.0001 | -0.782 | 0.0001 | 0.454 | 0.023 | -0.796 | 0.0001 | -0.771 | 0.0001 | -0.588 | 0.002 | 0.830 | 0.0001 |
| Male longevity | -0.844 | 0.001 | -0.233 | 0.262 | -0.547 | 0.005 | 0.236 | 0.255 | -0.490 | 0.013 | -0.581 | 0.002 | -0.105 | 0.619 | 0.548 | 0.005 |
| Female longevity | -0.947 | 0.0001 | -0.609 | 0.001 | -0.783 | 0.0001 | 0.585 | 0.002 | -0.759 | 0.0001 | -0.769 | 0.0001 | -0.521 | 0.008 | 0.874 | 0.0001 |


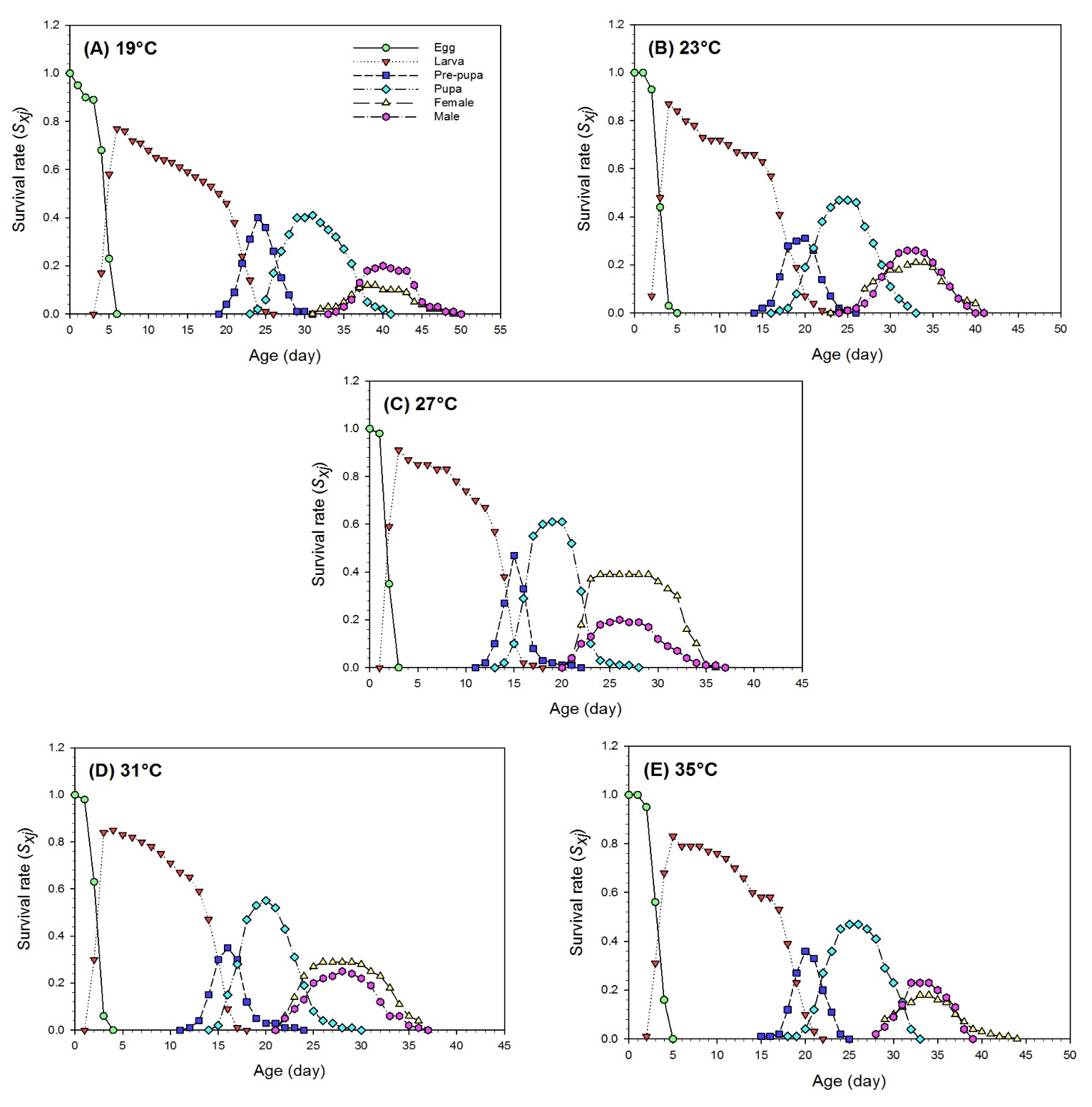


**Fig 2.** Age-stage-specific survival value (*Sxj*) of *Chrysodeixis includens* (SBL) fed on artificial diet reared at five different temperature regimes.


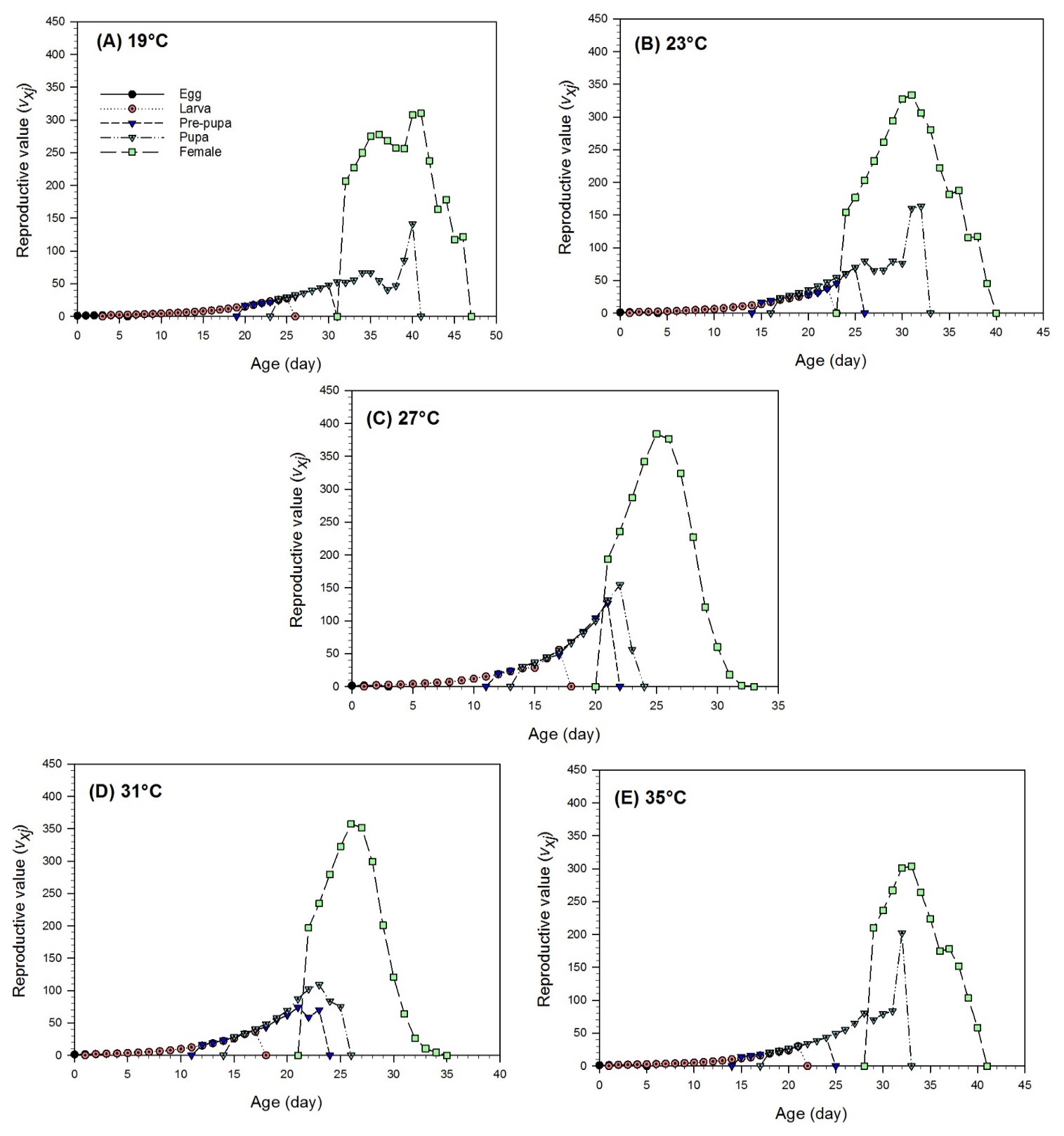


**Fig 3.** Age-stage-specific reproductive value (*v_xj_*) of *Chrysodeixis includens* (SBL) fed on an artificial diet reared at five different temperature regimes.


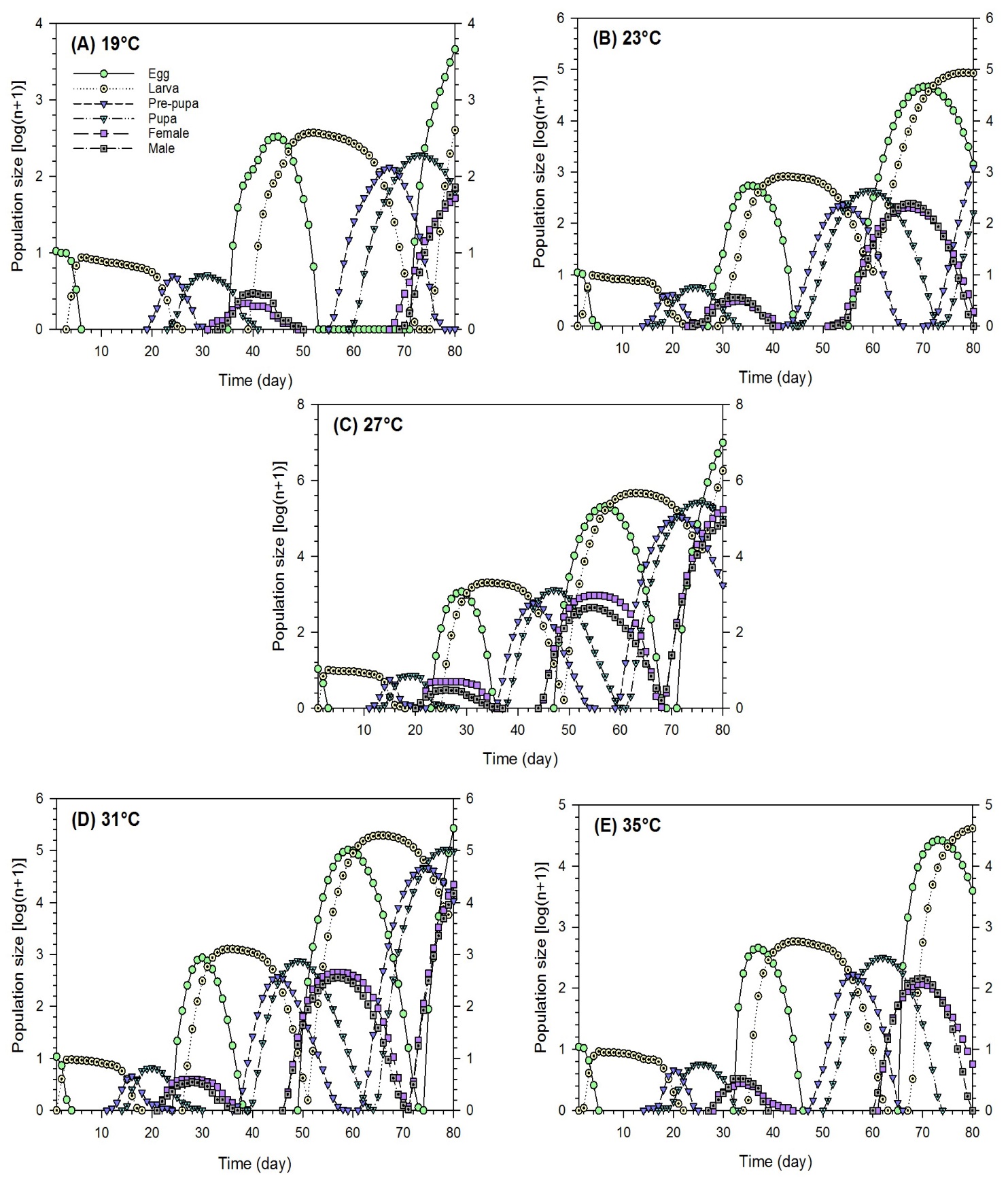


**Fig 4.** Projected population size of *Chrysodeixis includens* (SBL) fed on an artificial diet reared at five different temperature regimes.
